# The pedicle screw accuracy using a robotic system and measured by a novel three-dimensional method

**DOI:** 10.1186/s13018-023-04206-5

**Published:** 2023-09-20

**Authors:** Marcelo Oppermann, Vahagan Karapetyan, Shaurya Gupta, Joel Ramjist, Priscila Oppermann, Victor X. D. Yang

**Affiliations:** 1 Department of Clinical Neurological Science, Schulich School of Medicine and Dentistry, Western University, London, ON Canada; 2 Department of Electrical Computer and Biomedical Engineering, Toronto Metropolitan University, Toronto, ON Canada

**Keywords:** Pedicle screw, Robotic, Accuracy, Spatial 3D

## Abstract

Robotics in medicine is associated with precision, accuracy, and replicability. Several robotic systems are used in spine surgery. They are all considered shared-control systems, providing "steady-hand" manipulation instruments. Although numerous studies have testified to the benefits of robotic instrumentations, they must address their true accuracy. Our study used the Mazor system under several situations and compared the spatial accuracy of the pedicle screw (PS) insertion and its planned trajectory. We used two cadaveric specimens with intact spinal structures from C7 to S1. PS planning was performed using the two registration methods (preopCT/C-arm or CT-to-fluoroscopy registration). After planning, the implant spatial orientation was defined based on six anatomic parameters using axial and sagittal CT images. Two surgical open and percutaneous access were used to insert the PS. After that, another CT acquisition was taken. Accuracy was classified into *optimal, inaccurate and unacceptable* according to the degree of screw deviation from its planning using the same spatial orientation method. Based on the type of spatial deviation, we also classified the PS trajectory into 16 pattern errors. Seven (19%) out of 37 implanted screws were considered *unacceptable* (deviation distances > 2.0 mm or angulation > 5°), and 14 (38%) were inaccurate (> 0.5 mm and ≤ 2.0 mm or > 2.5° and ≤ 5°). CT-to-fluoroscopy registration was superior to preopCT/C-arm (average deviation in 0.9 mm vs. 1.7 mm, respectively, *p* < 0.003), and percutaneous was slightly better than open but did not reach significance (1.3 mm vs. 1.7 mm, respectively). Regarding pattern error, the tendency was to have more axial than sagittal shifts. Using a quantitative method to categorize the screw 3D position, only 10.8% of the screws were considered unacceptable. However, with a more rigorous concept of inaccuracy, almost half were non-optimal. We also identified that, unlike some previous results, the O-arm registration delivers more accurate implants than the preopCT/C-arm method.

## Introduction

Spine robotics is growing in importance and usage. Theoretically, they provide accuracy and replicability while accounting for patient-specific anatomical characteristics. Today’s systems are considered shared-control robots, providing "steady-hand" manipulation instruments while the surgeon controls the remainder of the procedure [1].

Numerous studies have addressed the benefits of robotics over other insertion techniques by linking the extent and rates of screw breaching [2–4]. Still, none have focused on how accurate the final trajectory is relative to its planning.

Our study compared the spatial accuracy of PS insertion along a planned trajectory between different registration methods and surgical access. Specific distances and angles between the PS and surrounding anatomical landmarks were measured for both planned trajectories and inserted screws, using preoperative and postoperative CT images, respectively.

The main objective of this cadaveric spine project was to define which registration method and surgical access showed the slightest deviation between the surgical plan and the final result. In addition, we investigated if there was a pattern of error for screw placement between the various robot-assisted methods, such as an average tendency to shift medially or superiorly.

## Materials and methods

### Subjects

This project used two cadaveric human samples (Science Care, Phonix—USA) with the entire spine from C7 to S1 (19 vertebrae each). A preoperative CT scan image (GE Discovery LS, Boston—USA) was taken in both spines to check the integrity of the bone and the posterior spine soft tissue. The specific image protocol used in the preoperative CT (zero-degree gantry angle and 0.625 mm slice thickness) was the same for the preopCT/C-arm registration method. Three screw dimensions were selected (4.5 × 45 mm, 5.5 × 45 mm and 6.5 × 50 mm—Voyager 5.5/60, Medtronic, Minneapolis—USA). This specific implant model can fit both open or percutaneous surgical methods. The robotic system used (Mazor X) was recently acquired, the first in Canada.

### Robotic setup

The setup process for Mazor X has been described elsewhere [5]. Briefly, it involves three steps: (1) image registration, (2) screw planning, and (3) robotic navigation. In the intraoperative O-arm method, the CT scanned the spine and a tracking device connected to the robotic arm. By including a coordinate marker with the spine in the same series, the robot can identify the location of each vertebra according to the surgical arm. In the preopCT/C-arm technique, a *pre*operative CT scan is used to plan the procedure, but the tracking device is acquired through a separate *intra*operative fluoroscopy. The two images are merged, allowing the robotic arm to move under continuous navigation. Ultimately, both methods can guide screw insertion for up to six vertebral levels, depending on the patient's anatomy and spine region.

### Study design

Two human cadaveric specimens, "W" and "B," were used. The preopCT/C-arm method was tested under percutaneous and open surgical access, while the O-arm method was only tested using percutaneous access. Screws were inserted in sequence from cranial to caudal vertebral levels. The robotic technique and implant dimensions are described in Table 1. After completion, cadavers were re-scanned using the same CT equipment and protocol.

**Table 1:** Implants sizes and techniques for each cadaver

Screw error patterns on axial and sagittal planes				
Vertebra	Cadaver W		Cadaver B	
	Screw Size	Technique	Screw Size	Technique
C7	4.5 × 35	O-Arm/Perc	4.5 × 35	O-Arm/Perc
T1	4.5 × 35	O-Arm/Perc	4.5 × 35	O-Arm/Perc
T2	5.5 × 45	O-Arm/Perc	4.5 × 35	O-Arm/Perc
T3	5.5 × 45	O-Arm/Perc	4.5 × 35	O-Arm/Perc
T4	5.5 × 45	Preop CT/Perc	4.5 × 35	O-Arm/Perc
T5	5.5 × 45	Preop CT/Perc	4.5 × 35	O-Arm/Perc
T6	5.5 × 45	Preop CT/Perc	5.5 × 45	Preop CT/Perc
T7	5.5 × 45	Preop CT/Perc	5.5 × 45	Preop CT/Perc
T8	5.5 × 45	Preop CT/Perc	5.5 × 45	Preop CT/Perc
T9	6.5 × 50	Preop CT/Perc	5.5 × 45	Preop CT/Perc
T10	6.5 × 50	Preop CT/Perc	5.5 × 45	Preop CT/Perc
T11	6.5 × 50	Preop CT/Perc	6.5 × 50	Preop CT/Perc
T12	6.5 × 50	Preop CT/Open	6.5 × 50	Preop CT/Open
L1	6.5 × 50	Preop CT/Open	6.5 × 50	Preop CT/Open
L2	6.5 × 50	Preop CT/Open	6.5 × 50	Preop CT/Open
L3	6.5 × 50	Preop CT/Open	6.5 × 50	Preop CT/Open
L4	6.5 × 50	Preop CT/Open	6.5 × 50	Preop CT/Open
L5	6.5 × 50	Preop CT/Open	6.5 × 50	Preop CT/Open
S1	6.5 × 50	Preop CT/Open	6.5 × 50	Preop CT/Open

PreopCT/C-arm was tested using percutaneous (perc) or open surgical access. O-arm was tested only using percutaneous access

### Imaging analysis

DICOM formatted preoperative and postoperative images were analyzed on a personal computer using the Mazor system software (Mazor X Version 5.0.1.77, Caesarea—Israel). For each screw trajectory, specific distances and angles were calculated concerning anatomical landmarks on axial and sagittal planes. Each plane had two distances (proximal and distal to the screw head) and one angular measurement.

**Fig. 1:**
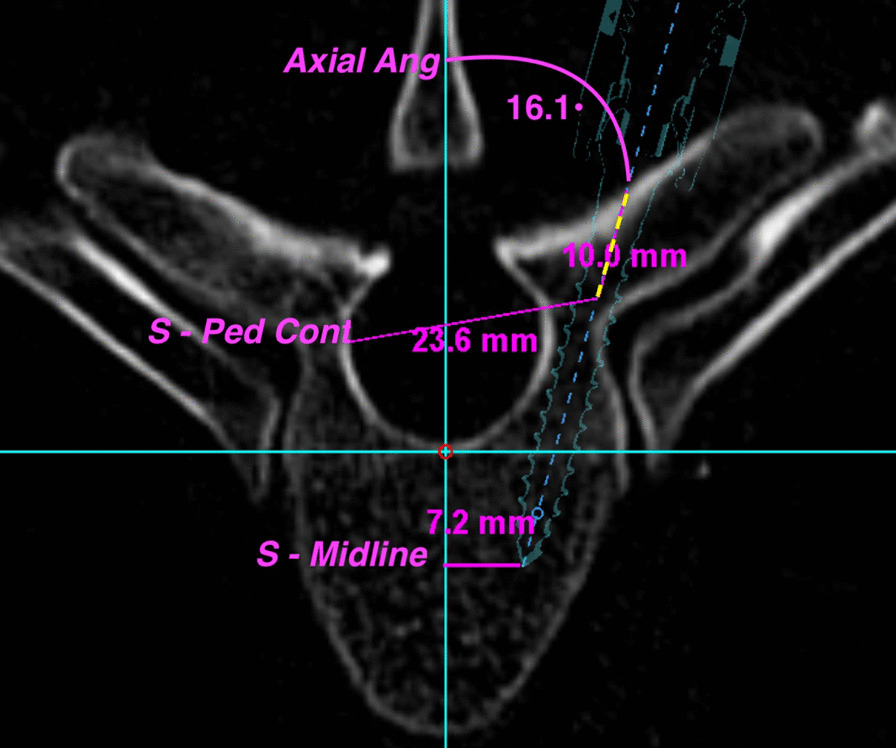
Shows the axial parameters; 7.2 mm represents the distance between the midpoint of the vertebral canal line and the tip of the screw (S—midline), 23.6 mm represents the distance between two points, the most convex area of the contralateral pedicle and 10 mm from the entry point on the screw projection (S—Cont Ped). The angle (16.1°) shows the axial angulation of the screw with the midpoint line (Ax Ang)

Considering the axial plane (Fig. 1), the proximal measurement involved the distance between the proximal screw and the contralateral pedicle. The proximal screw was defined as a point where the screw was one centimetre deep from its entrance. The contralateral pedicle point was considered the far-lateral point of the spinal canal, which coincided with the medial surface of the contralateral pedicle (*S—Cont Ped*). For the distal axial measurement, we first needed to identify an axial midline, which can be challenging given the natural asymmetry of vertebral processes. We, therefore, opted to use the center point of the spinal canal and vertebral body, through which we defined a *central vertebral line.* The distance from the tip of the screw to the *central vertebral line* was then measured (*S—Midline*). Finally, we calculated the angle subtending the screw’s axial trajectory with the *central vertebral line* (*Ax Angle*).

**Fig. 2:**
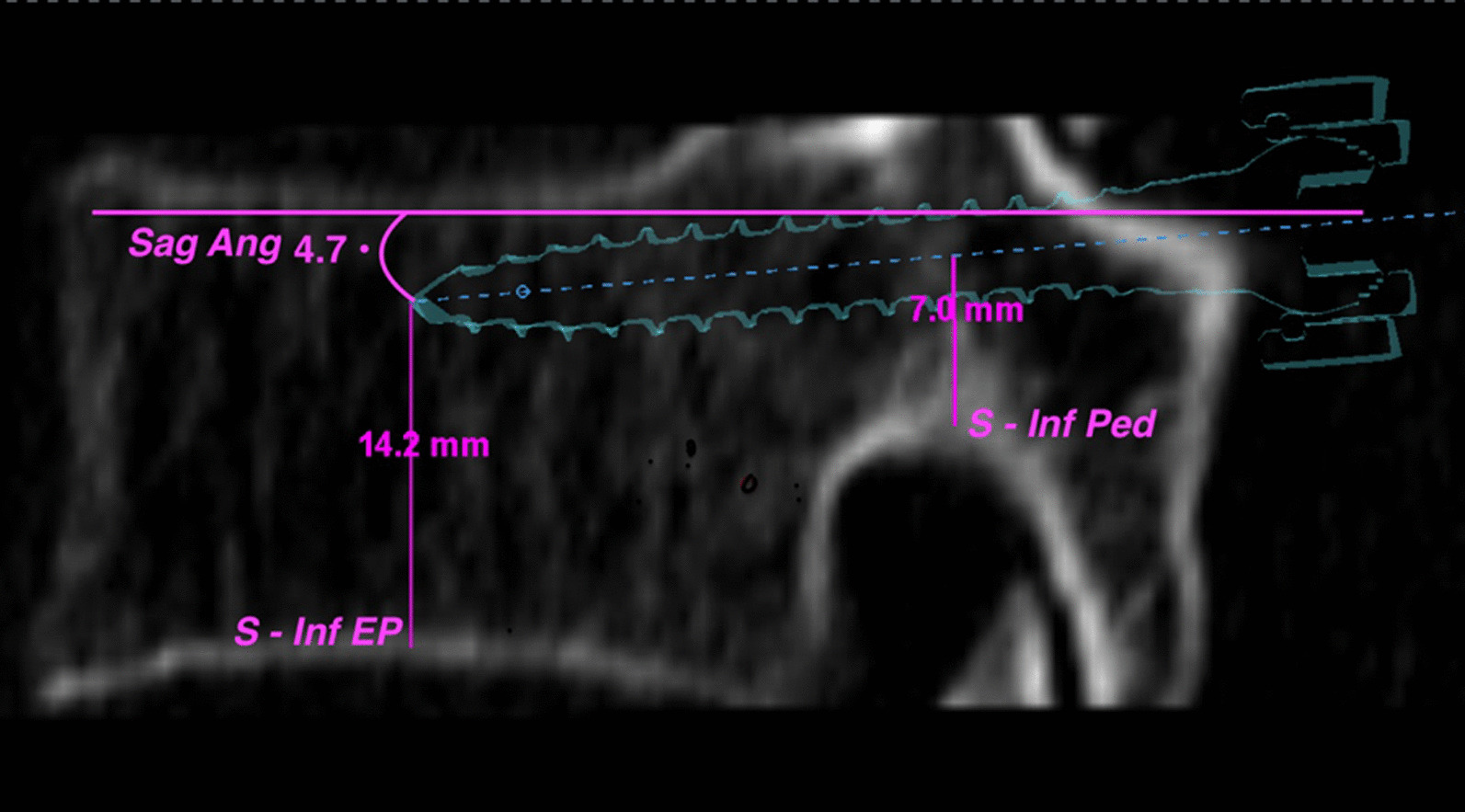
Shows the sagittal parameters; 14.2 mm represents the distance from the projection of the tip of the screw on the inferior end plate (S-Inf EP). The length of the center of the screw with its projection to the inferior pedicle cortical bone is represented by 7.0 mm here (S- Inf Ped). The angle (4.7°) shows the sagittal angulation of the screw with the inferior endplate representative line (Sag Ang)

On the sagittal plan (Fig. 2), the proximal distance measurement considered a straight line from the center of the screw to the cortical surface of the inferior pedicle (*S—Inf Ped*). The distal measure contemplated a line from the tip of the screw to the inferior endplate of the vertebral body (*S—Inf EP*). And the angular measurement subtended the angle between the screw trajectory and a line tangent to the inferior endplate (*Sag Ang*).

For each implanted screw, the arithmetic difference between the preoperative plan and the final screw position was calculated from the six above measures (four distances and two angles). This permitted us to identify superior-inferior and medial–lateral shifts between planned and actual screw trajectories. For example, in the axial plane, a positive value indicates a medial deviation and a negative value indicates a lateral deviation. While in the sagittal plane, a positive value is characterized by inferior variation and a negative value by superior deviation.

Accuracy was classified into three groups: *optimal, inaccurate and unacceptable*. *Optimal* was defined for the screws with planned and postop differences ≤ 0.5 mm for direct distance and ≤ 2.5° for angle. Differences of > 2.0 mm and > 5° would be deemed *unacceptable.* The *inaccurate* group involved screws where the final trajectory deviated beyond the limits of measurement but remained within the acceptable range (distance deviations > 0.5 mm and ≤ 2.0 mm, and angle deviations > 2.5° and ≤ 5°).

**Fig. 3:**
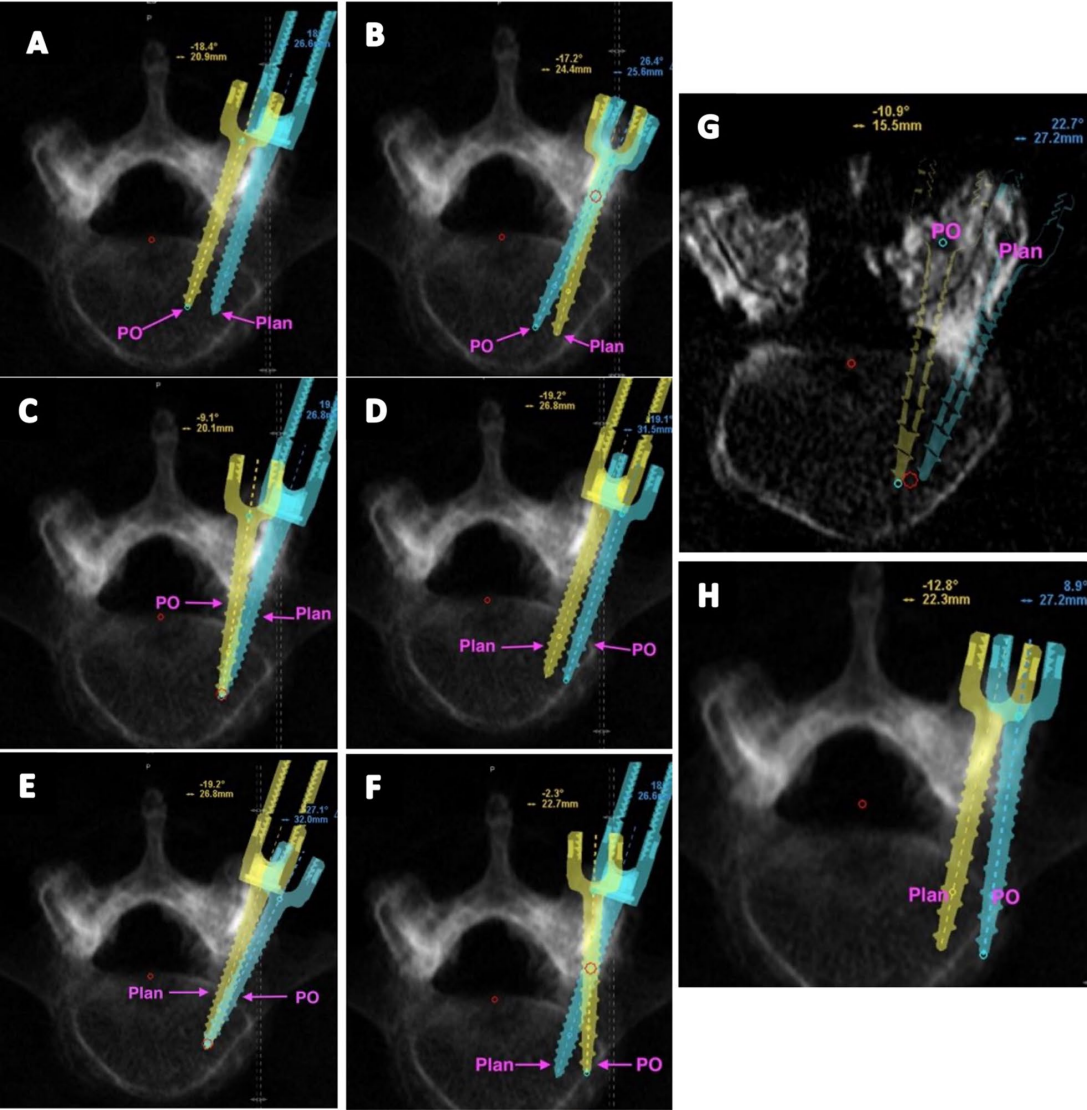
Axial screw error patterns based on planned (plan) axial trajectories and postoperative (PO) screws. **A** Symmetric medial deviation, **B** distal medialization, **C** proximal medialization, **D** symmetric lateral deviation, **E** proximal lateralization, **F** distal lateralization, **G** full medial deviation, and **H** full lateral deviation

**Fig. 4:**
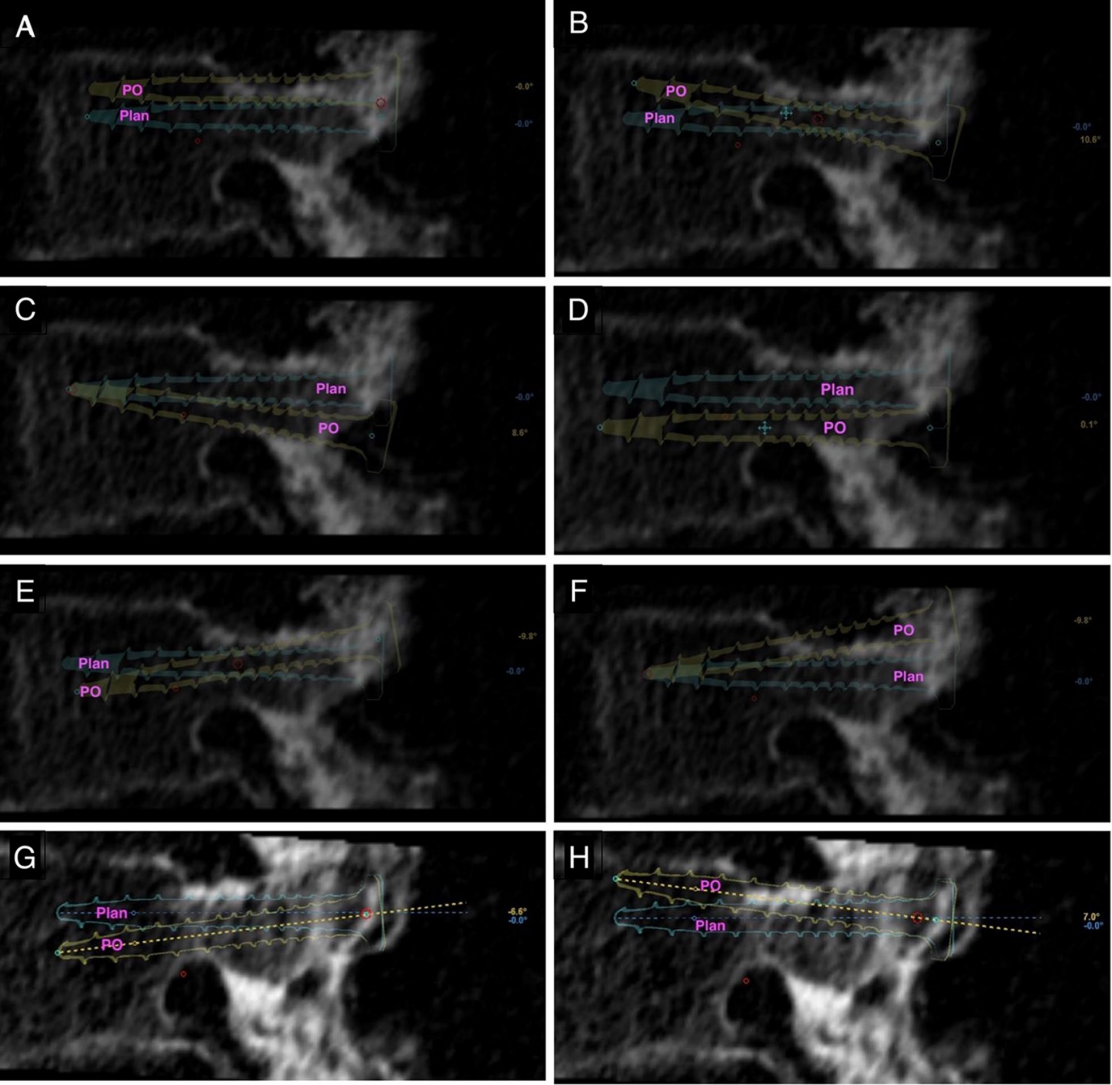
Sagittal screw error patterns based on planned (plan) sagittal trajectories and postoperative (PO) screws. **A** Symmetric superior deviation, **B** distal superior deviation, **C** proximal inferior deviation, **D** symmetric inferior deviation, **E** distal inferior deviation, **F** proximal superior deviation, **G** full inferior deviation, and **H** full superior deviation

All suboptimally placed screws (*inaccurate* and *unacceptable* groups) were classified according to their deviation pattern error into eight suboptimal trajectories. The patterns are illustrated in Fig. 3 (axial) and Fig. 4 (sagittal), and the classification criteria are detailed in Table 2.

**Table 2:** Screw error parameters

Screw error patterns on axial and sagittal planes									
Axial plane					Sagittal plane				
Screw pattern axial	Figure reference	Measured parameters			Screw pattern Sag	Figure reference	Measured parameters		
		S—Cont Ped	S—Midline	|Ax Angle|			S—Inf Ped	S—Inf EP	|Sag Angle|
Symmetric medial deviation	Picture 3A	> 0.5	> 0.5	< 2.5	Symmetric superior deviation	Picture 4A	< − 0.5	< − 0.5	< 2.5
Distal medialization	Picture 3B	− 0.5 < *x* < 0.5	> 0.5	> 2.5	Distal superior deviation	Picture 4B	− 0.5 < *x* < 0.5	< − 0.5	< 2.5
Proximal medialization	Picture 3C	> 0.5	− 0.5 < *x* < 0.5	> 2.5	Proximal inferior deviation	Picture 4C	> 0.5	− 0.5 < *x* < 0.5	> 2.5
Symmetric lateral deviation	Picture 3D	< − 0.5	< − 0.5	< 2.5	Symmetric inferior deviation	Picture 4D	> 0.5	> 0.5	< 2.5
Proximal lateralization	Picture 3E	< − 0.5	− 0.5 < *x* < 0.5	> 2.5	Distal inferior deviation	Picture 4E	− 0.5 < *x* < 0.5	> 0.5	> 2.5
Distal lateralization	Picture 3F	− 0.5 < *x* < 0.5	< − 0.5	> 2.5	Proximal superior deviation	Picture 4F	< − 0.5	− 0.5 < *x* < 0.5	> 2.5
Full medial deviation	Picture 3G	> 0.5	> 0.5	> 2.5	Full inferior deviation	Picture 4G	> 0.5	> 0.5	> 2.5
Full lateral deviation	Picture 3H	< − 0.5	< − 0.5	> 2.5	Full superior deviation	Picture 4H	< − 0.5	< − 0.5	> 2.5

Eight distinct patterns of screw placement error were identified along the axial (left) and sagittal (right) plane by measuring differences between planned preoperative and final postoperative screw trajectories. Axial deviations reference Fig. 3, and Sagittal deviations reference Fig. 4

### Statistics

Statistical analyses were completed in R (2022.07.2 + 576). Contingency tables were analyzed using Fisher’s exact test. To compare the performance of different pedicle screw insertion methods, the maximal distance deviation for each screw was compared across groups using the Kruskal–Wallis rank sum test. Post hoc pairwise comparisons were conducted using the Mann–Whitney *U* test. Benjamini–Hochberg correction was used for multiple comparisons, and significance was set at *p* < 0.05. Principal component analysis (PCA) was conducted to identify which measures contributed to the maximal variation in screw placement error.

## Results

### Subjects

Thirty-seven screws were inserted on the right side of the two cadaveric specimens. We could not insert the right S1 implant on cadaver W due to poor bone quality (low density) on that specific vertebra. Otherwise, all PS were inserted as planned (Table 1).

### Unacceptable screws

From all implanted screws, 7/37 (19%) were considered *unacceptable* (distance > 2.0 mm or angulation > 5°). On cadaver W, they were in the lumbar region (L3 and L4), while on cadaver B, they were in the lower thoracic (T10, T11, T12) and lumbosacral (L5 and S1) regions. Comparing the three insertion methods, the percutaneous O-arm performed best with no unacceptable implants. The percutaneous preopCT/C-arm method had the next best performance with only two unacceptable screw placements (15%), and the open preopCT/C-arm technique had the poorest performance with five unacceptable screws (38%). All seven unacceptable screws had significant deviations in the axial plane and predominantly in the lateral direction. At the same time, only 3/7 were deviated in the sagittal plane and predominantly in the inferior direction.

### Inaccurate screws

Inaccuracy was identified in 14/37 (38%) of the implants, where at least one of the measures deviated from the planned trajectory by > 0.5 mm and ≤ 2.0 mm for distances or > 2.5° and ≤ 5° for angulation. Sixteen screws had optimal results (43%). Both cadavers had a similar number of inaccurate implants (Table 3).

**Table 3:** Screw accuracy per level in both cadavers

Vertebra	Screws accuracy in both cadavers			
	Cadaver W		Cadaver B	
	Axial	Sagittal	Axial	Sagittal
C7	Proximal medialization	Optimal	Optimal	Optimal
T1	Distal lateralization	Optimal	Proximal medialization	Distal inferior deviated
T2	Optimal	Optimal	Optimal	Optimal
T3	Optimal	Optimal	Optimal	Optimal
T4	Optimal	Optimal	Optimal	Optimal
T5	Optimal	Optimal	Optimal	Distal inferior deviated
T6	Optimal	Optimal	Optimal	Optimal
T7	Full medial deviated	Optimal	Optimal	Optimal
T8	Optimal	Optimal	Symmetric medial deviated	Symmetric inferior deviated
T9	Symmetric medial deviated	Optimal	Symmetric medial deviated	Optimal
T10	Symmetric medial deviated	Symmetric inferior deviated	Full medial deviated	Symmetric inferior deviated
T11	Optimal	Optimal	Unacceptable	Unacceptable
T12	Optimal	Symmetric inferior deviated	Unacceptable	Unacceptable
L1	Optimal	Optimal	Optimal	Optimal
L2	Unacceptable	Unacceptable	Symmetric medial deviated	Optimal
L3	Unacceptable	Unacceptable	Optimal	Symmetric inferior deviated
L4	Full lateral deviated	Symmetric inferior deviated	Optimal	Optimal
L5	Symmetric lateral deviated	Optimal	Full medial deviated	Optimal
S1	None	None	Symmetric medial deviated	Optimal
Total/plane	7/15	3/15	7/16	5/16
Total/screw	8/15		9/16	

The total number of inaccurate implants is detailed below (excluding unacceptable screw placements). The total/plane considers only the anatomic plane, whereas the total/screw shows the total screws that were inaccurate in at least one plane

Using the screw error pattern classification (Table 2), we found that Symmetric Medial Deviation (4/14) was the most common error in the axial plane, and Symmetric Inferior Deviation (5/14) was the predominant pattern in the sagittal plane. Comparing the maximal screw distance deviations in the axial vs. sagittal planes, the extent of error within each plane was similar (median and IQR = 1.5 mm [0.93, 1.7] and 0.85 mm [0.40, 1.43], respectively; Mann–Whitney U *W* = 132.5, *p* = 0.12), although there was a tendency for more errors to be in the axial plane. When combined with *Unacceptable* screws, this difference became significant, with more errors appearing in the axial plane (1.7 mm [1.0, 2.4]) compared to the sagittal plane(0.9 mm [0.4, 1.7]; *W* = 325.5, *p* = 0.009).

### Surgical methods

The two registration methods (O-arm and preopCT/C-arm) and two surgical approaches (open and percutaneous) were analyzed separately and in combination. Using the maximum deviation in distance measurements between the preoperative plan and the final trajectory for each screw, the O-arm (*N* = 11, median and IQR = 0.9 [0.8, 1.25] mm) and preopCT/C-arm (*N* = 26, 1.7 [1.2, 2.0] mm) image registration methods were compared to one another. The former was significantly more accurate (*W* = 52.5, *p* = 0.003). When only the surgical access method was considered, the percutaneous approach had slightly better outcomes than the open approach (1.3 [0.88, 1.7] mm s 1.7 [1.2, 2.6] mm), but results did not reach significance (*W* = 210, *p* = 0.09).

**Fig. 5:**
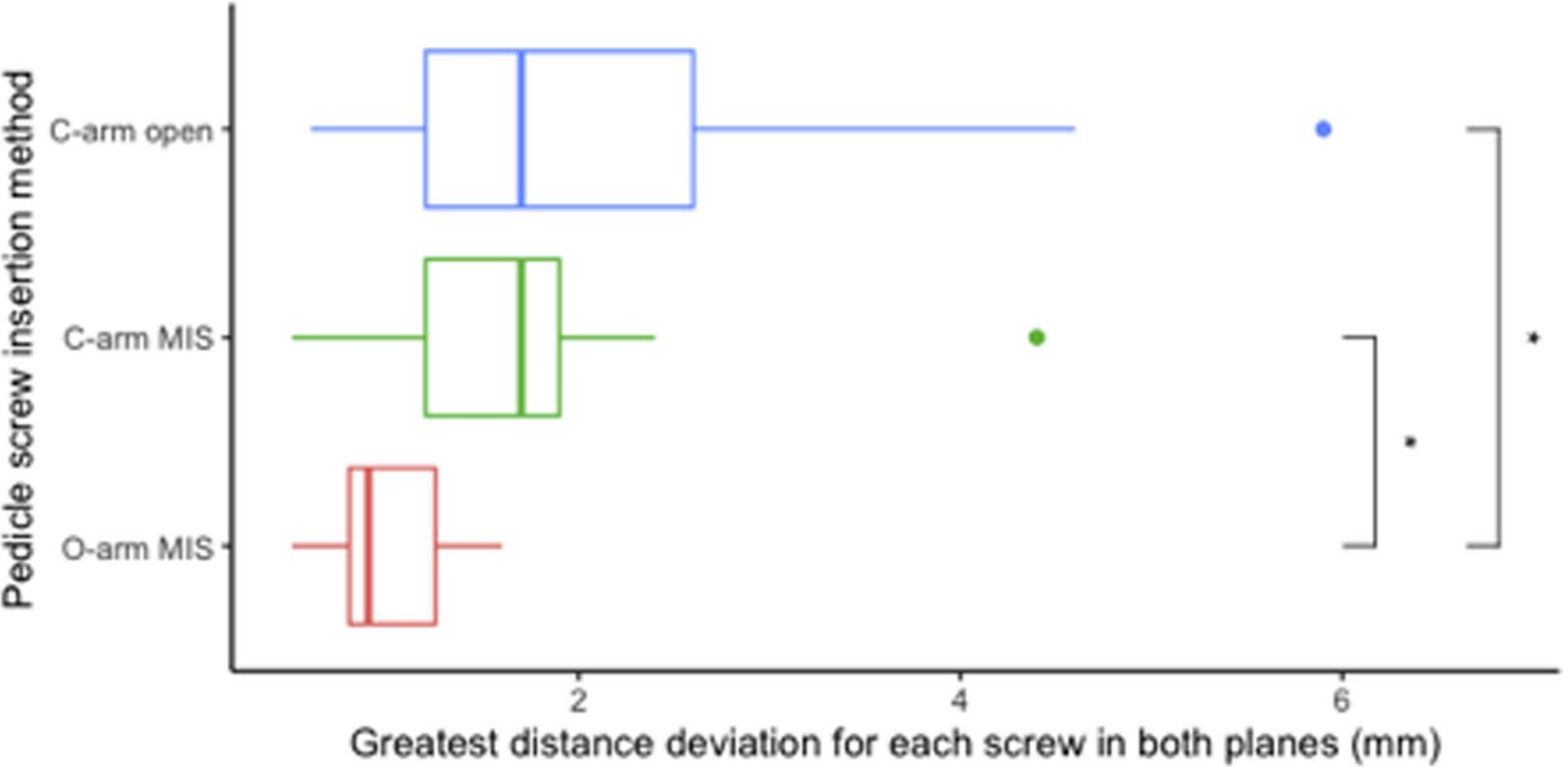
The greatest distance deviation for each pedicle screw from planned trajectories across axial and sagittal planes, grouped by different insertion methods. Plots are median, and IQR and outliers are beyond 1.5 × IQR. The o-arm insertion method resulted in smaller errors than both C-arm insertion methods (stars indicate *p* < 0.05, Benjamini–Hochberg correction for multiple comparisons)

Looking at the performance of registration methods and surgical approaches in combination (Fig. 5), the percutaneous O-arm was superior (*N* = 11, 0.9 [0.8, 1.25] mm), followed by the percutaneous preopCT/C-arm (*N* = 13, 1.7 [1.2, 1.9] mm), and finally, open preopCT/C-arm (*N* = 13, 1.7 [1.2, 2.6] mm). Groupwise analysis with the Kruskal–Wallis rank sum test found these differences significant (*Χ*^2^(2) = 9.18, *p* = 0.01). A post hoc pairwise study using the Mann–Whitney *U* test and Benjamini–Hochberg correction for multiple comparisons found the percutaneous O-arm method to perform significantly better than the percutaneous and open preopCT/C-arm methods (*p* = 0.02). The two preopCT/C-arm methods performed similarly (*p* = 0.61).

**Fig. 6:**
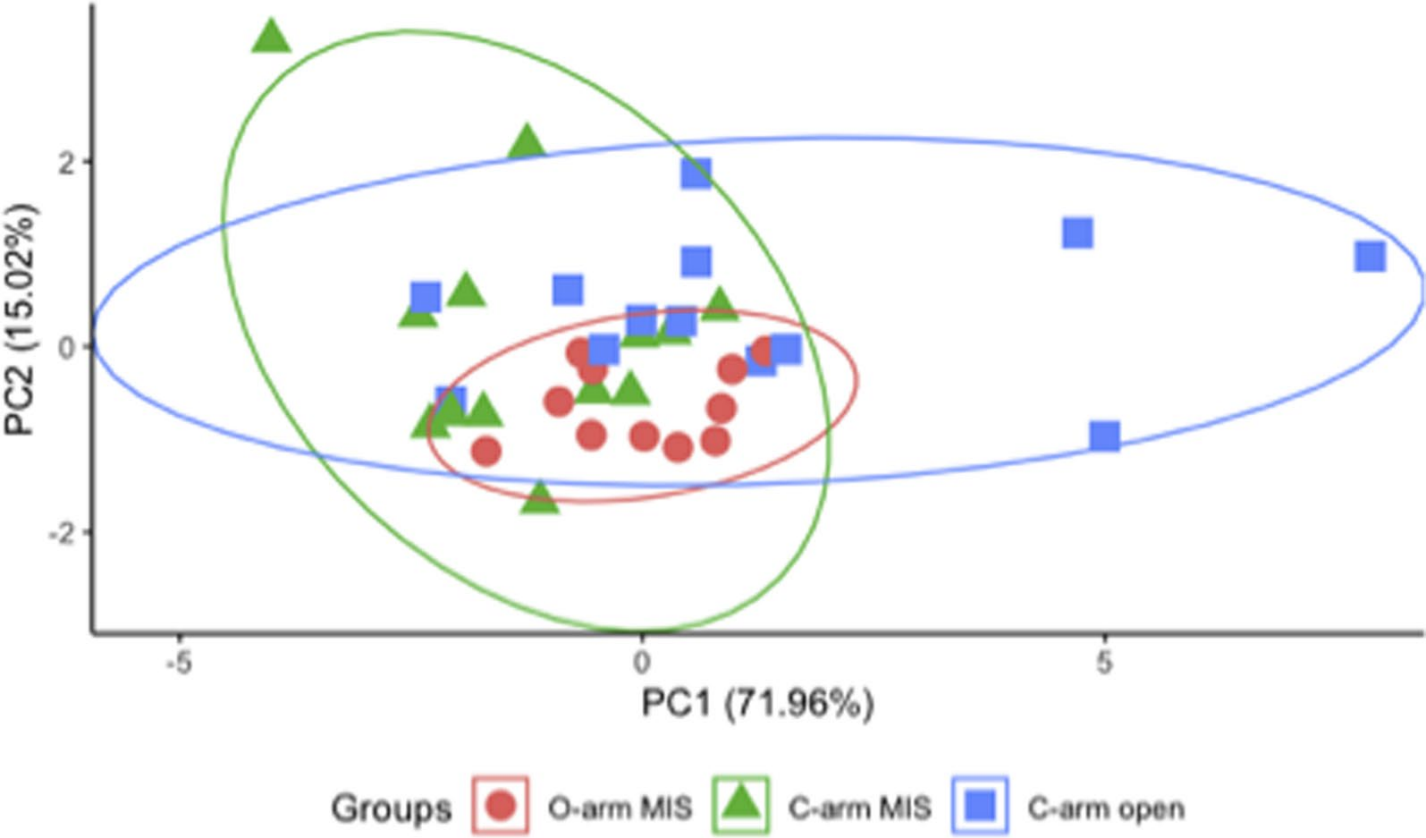
Principal component analysis of distance deviations for all pedicle screws. The first two principal components account for 87% of all deviations in distance measurements between planned and final screw trajectories. Data have been grouped by insertion technique, and ellipses indicate 95% confidence bounds on Gaussian approximations for each group

Considering all four distance deviations for each screw, we conducted a principal component analysis (PCA), identifying linear combinations of error that most contributed to inaccurate screw placement. The two main components plotted in Fig. 6 encompass 87% of the total deviation in distance measurements between the planned and final screw trajectories. For PC1, 95% of the contribution was from errors in the axial plane, with approximately equal influence from both axial measures. For PC2, 32% of the contribution was from the *Screw—Pedicle* distance in the sagittal plane, with a further 51% from the two axial measures. These results reinforce the prior findings that the most significant errors in screw placement are in the axial plane. By grouping screws according to the insertion method and using a Gaussian approximation for the error distribution along principal components, Fig. 6 also demonstrates the smaller overall error in final screw trajectories for the percutaneous O-arm technique, as compared to both preopCT/C-arm methods.

## Discussion

Although robotic spine systems can offer many advantages, including reduced radiation exposure and decreased invasiveness, the increment in precision remains essential in favor of their use.

In the spine, there are several methods to measure implant accuracy. Some use the criteria of "in" or "out" [6], others use pedicle breach analysis [7–9], and yet others attempt to quantify the amount of facet joint violation [10]. Although these approaches can correlate clinically with patient outcomes, they do not clearly define accuracy. It is reasonable to use the "breach" concept (*Gertzbein–Robbins* scale) to check accuracy when the implant is inserted in a broad pedicle (> 7 mm). In this context, for a 5-mm-diameter PS to still be considered acceptable (< 2 mm breach), it is necessary to have no more than a 3 mm shift from the original trajectory. However, in a narrow pedicle (< 4 mm), the same 3 mm shift is not an appropriate measure. This qualitative method is vague and cannot be applied to all patients and spinal levels. In discordance with this method, we used a spatial orientation or quantitative approach to compare PS accuracy in the current study.

Our experiment measured preoperative and postoperative screw distances and angles in two anatomic planes. A variation of this method has been used before [11, 12], but we added new features which offer several advantages. To begin with, ours is the first to assess the performance of robotic instrumentation. Second, comparing planned preoperative trajectories with postoperative measurements allowed us to extrapolate *Postoperative Screw Error Patterns*. This involved using anatomically derived measures of screw position, which described 3D screw trajectories, and analyzing how final screw placements deviated from the planned trajectories. By classifying trajectory errors, it allows robot calibration, avoiding future deviances. Another benefit of our measurement methodology is that we used the contralateral pedicle as one of the measures in the axial plane, as opposed to the more commonly used ipsilateral pedicle [7–9]. Several authors described how the implants' metallic artifact sometimes obscures the screw boundaries, making pedicle breach assessment almost impossible [13]. None of our screw's metallic artifacts tampered with the measurement using the contralateral pedicle as a reference.

The discussion of how to define pedicle screw accuracy and its importance is sparse in the literature. Rampersaud et al. are among the few groups addressing this question [14]. In their paper, accuracy requirements differ depending on the spine level. These requirements often exceed the accuracy of current image-guided surgical systems based on clinical utility errors reported in the literature. In their conclusion, the authors state that maximum permissible translational/rotational error tolerances ranged from 0.0 mm/0.0° at T5 to 3.8 mm/12.7° at L5. In our cadavers, we divided the non-optimal PS into two classes: *Unacceptable* (> 2.0 mm for distance and > 5° for angulation) and *Inaccurate* (between > 0.5 mm and ≤ 2.0mmn and > 2.5° and ≤ 5°, respectively). Ideally, a robotic system should produce no noticeable screw trajectory errors, but technology has yet to mature. In addition, it is challenging to measure screw trajectories from CT images without a repeatability error of less than 0.5 mm or 2.5°. To minimize this error, we measured preoperative and postoperative images using the same planning software. A third-party application was considered less consistent.

A few others have used the quantitative concept to measure their implants utilizing direct distances and angles to compare planned and postoperative images [11, 12, 15]. *Ortel *et al. quantified only the axial angle using a "midsagittal line" passing in the middle of the vertebra, taking the spinous process as a reference [15]. This line is not always straightforward, especially in cases where the vertebrae are asymmetrical. Instead, we used the vertebral canal and body as a reference; this method was less affected by anatomical irregularities. *Kleck and al*. also measured the axial angle but included a direct distance from the entry point to the tip of the screw [11]. Although valid, this does not give enough information to fully represent the screw position in both preoperative and postoperative images. *Guha *et al*.* measured both axial and sagittal angles [12]. They included the distance between the screw entry point and the mid-sagittal line (bisecting the vertebral body, spinal canal, and spinous process). Although their technique provides some information about the 3D orientation of the screw, it does not specify the screw's tip coordinates inside the vertebra. We demarcated the precise spatial orientation inside the vertebra by considering the screw’s proximal and distal directions. The 3D anatomical definition was crucial to characterize the screw accuracy.

In our study, the incidence of unacceptable screws in our sample (10.8%) was consistent with other studies [3, 4, 16–19], which used the *Gertzbein–Robbins* scale for measuring accuracy [8]. However, our data added new insights when labeling implants as inaccurate. Based on our results, only 43.2% of the PS were found to precisely replicate the preoperative plan, with the remaining placed screws being either inaccurate (37.8%) or wholly unacceptable (18.9%). This is the first time a study has exposed the limits of the current robotic technology, as suggested by some authors [14]. In addition, our study protocol allowed us to analyze the accuracy of different registration techniques (O-arm and preopCT/C-arm).

In numerous articles, o-arm accuracy has been compared against fluoroscopy; overall, it performs better [16, 20–23]. However, its efficacy in robotic surgery is still to be defined. Two studies tested accuracy using the *Gertzbein–Robbins* scale and found no difference between the O-arm and preopCT/C-arm registration techniques for unacceptable screws (> grade I) [5, 24]. Our results had a different outcome. In our series, the O-arm was superior to the preopCT/C-arm. It is essential to point out that the upper cervical screw angle is particularly challenging for any technique, navigated or not. They are usually very cranial (high sagittal) and medial (high axial) in angle. Moreover, because of the high sagittal degree, the navigated dynamic reference array (sphere trackers) can be easily blocked from the navigation camera. So, theoretically, the most challenged screws were placed using the O-arm method and still yielded more accurate results. The reasons may be linked to the acquisition process. The preoopCT/C-arm method requires imaging fusion and matching, and the algorithm behind it depends on bone density. So the merging process between the preopCT and the C-arm images may be affected by osteoporosis. In fact, some authors have described that the robotic system cannot recognize the vertebral anatomy from the poor-quality intraoperative fluoroscopic [2, 19]. In our sample, the S1 screw of cadaver W was not inserted for this reason. Based on these data, viewing the O-arm as the superior method is not difficult, but a confirmatory investigation is required.

In terms of surgical access, apart from the advantages or disadvantages of the two techniques, some scientific studies claim that open exposure can tamper with screw accuracy when soft tissue retraction is not optimal or if soft tissues encumber the surgical robotic arm [2, 3, 25]. Our results comparing percutaneous and open approaches may have been influenced by inadequate exposure to robotic standards. When considering all screws, irrespective of the registration method, the less invasive procedure (percutaneous) had better performance. For example, we noticed inappropriate muscular exposure for the L3 and L4 screws during the cadaver W surgical procedure. This may have interfered with the postoperative result of these two implants. In part, our results were similar to *Kantelhardt *et al*.* [25]. Although both open and percutaneously placed screws have not generally differed in their study, some significance was found toward less invasiveness, but only for implants entirely inside the bone. In our case, the overall O-arm superiority may have influenced the better percutaneous performance. Unfortunately, we did not use open access with the O-arm to confirm these results. Nevertheless, the results were similar when both open and percutaneous access were compared for preopCT/C-arm registration.

Our study has several limitations. Despite what some consider a positive factor [8] lacks inter-investigator variability. Second, the small sample size limits the broad generalizability of the results, but by adding several measurements per screw, our results reached significance. It is essential to mention that although our group has approximately 150 h of robotic cadaver training, our learning curve has not yet plateaued.

## Conclusion

Our robotic cadaveric study used a quantitative method to categorize the screw 3D position. With that, the preoperatively planned and postoperative screw locations could be compared. Our methodology was designed to help elaborate a screw error pattern classification. Moreover, it allowed grouping.

In our casuistic, only 10.8% of the screws were considered unacceptable; conversely, if the new, most rigorous concept of inaccuracy was added, almost half were non-optimal. We also identified that, as opposed to some previous results, the O-arm registration delivers more accurate implants than the preopCT/C-arm method.

Although our sample is restricted to only 37 screws, the results may suggest changes in the next generation of robotic systems. The future designs compel new methods to improve accuracy and must offer tools to correct repeated erratic patterns.

## Data Availability

The data can be requested directly from the main author’s (MO) email.
